# Projecting the Potential Distribution Areas of *Ixodes scapularis* (Acari: Ixodidae) Driven by Climate Change

**DOI:** 10.3390/biology11010107

**Published:** 2022-01-10

**Authors:** Lu Zhang, Delong Ma, Chao Li, Ruobing Zhou, Jun Wang, Qiyong Liu

**Affiliations:** 1 State Key Laboratory of Infectious Disease Prevention and Control, Collaborative Innovation Center for Diagnosis and Treatment of Infectious Diseases, National Institute for Communicable Disease Control and Prevention, Chinese Center for Disease Control and Prevention, Beijing 102206, China; zhangluicdc@163.com (L.Z.); madelong97@163.com (D.M.); lichaoicdc@163.com (C.L.); zrb9610@126.com (R.Z.); wangjun@icdc.cn (J.W.); 2 School of Public Health and Health Management, Shandong First Medical University and Shandong Academy of Medical Sciences, Jinan 250117, China; 3 Shandong University Climate Change and Health Center, School of Public Health, Shandong University, Jinan 250012, China

**Keywords:** *Ixodes scapularis*, potential distribution, MaxEnt, climate change

## Abstract

**Simple Summary:**

*Ixodes scapularis* is a vector of tick-borne diseases. Climate change is one of the main factors affecting the distribution of *I. scapularis*. We used the maximum entropy model to project the potential distribution and future trends of *I. scapularis*. The potential suitable area of *I. scapularis* is dynamically changing in the context of climate change and precipitation in May makes the greatest contribution to such expansion.

**Abstract:**

*Ixodes scapularis* is a vector of tick-borne diseases. Climate change is frequently invoked as an important cause of geographic expansions of tick-borne diseases. Environmental variables such as temperature and precipitation have an important impact on the geographical distribution of disease vectors. We used the maximum entropy model to project the potential geographic distribution and future trends of *I. scapularis*. The main climatic variables affecting the distribution of potential suitable areas were screened by the jackknife method. Arc Map 10.5 was used to visualize the projection results to better present the distribution of potential suitable areas. Under climate change scenarios, the potential suitable area of *I. scapularis* is dynamically changing. The largest suitable area of *I. scapularis* is under SSP3-7.0 from 2081 to 2100, while the smallest is under SSP5-8.5 from 2081 to 2100, even smaller than the current suitable area. Precipitation in May and September are the main contributing factors affecting the potential suitable areas of *I. scapularis*. With the opportunity to spread to more potential suitable areas, it is critical to strengthen surveillance to prevent the possible invasion of *I. scapularis*.

## 1. Introduction

*Ixodes scapularis* (*I. scapularis*) is a vector of many tick-borne diseases [1]. In the United States, for example, *I. scapularis* is a vector of seven human pathogens [2]. Climate change is believed to be a factor in the increased incidence of tick-borne diseases [3,4]. Since Lyme disease was first described in the 1970s in the United States, its geographical range has gradually expanded from northeastern to western and southern, and even to Eurasia and other regions [5,6]. Evidence is mounting that climate change affects the spread of Lyme disease [7]. As the main vector of Lyme disease, *I. scapularis* is also affected by climate change. Studies have found that the geographic distribution of *I. scapularis* and the pathogens it carrys has been expanding over the past 20 years [2]. The impact of climate change on tick vectors and diseases is expected to be profound [8].

Currently, climate change is one of the major international concerns. The Intergovernmental Panel on Climate Change (IPCC) published Shared Socioeconomic Pathways (SSPs) [9], in 2010, which were developed on the basis of representative concentration pathways (RCPs) to quantitatively describe the relationship between climate change and socioeconomic development paths and reflect future climate change adaptation and mitigation challenges for society. Many researchers have used SSPs to simulate future climate change to project suitable areas of different species [10,11,12,13,14]. It is critical to understand the impact of climate change on vector distribution, which can provide a theoretical basis for the prevention and control of vector-borne diseases.

Species distribution models are widely used in various research fields. The most common species distribution models include GARP, EnFA, BIOCLIM, DOMAIN, MaxEnt, etc. [15]. The MaxEnt model is the ecological model with the best prediction effect at present. Compared with other models, its prediction is more consistent with the real world [16]. The MaxEnt model can accurately predict the potential distribution of species through the data of the species’ distribution points and environmental variables, providing an important basis for scientific research. Even a few distribution points can be accurately predicted as compared with other models [17]. The MaxEnt model can self-check the simulated prediction by using the receiver operating characteristic curve (ROC) of the jackknife test. The jackknife method has also been selected to measure the contribution of each environmental variable and to judge the different effects of different environments on species distribution [18,19]. 

This study used the MaxEnt model, based on the actual distribution sites of *I. scapularis* and environmental variables, to project the potential distribution of *I. scapularis* internationally and to find the main contributing environmental variables under future climate scenarios.

## 2. Materials and Methods

### 2.1. Software and Geographic Data

Maxent software (version 3.4.1, https://biodiversityinformatics.amnh.org/, accessed on 23 May 2021) was used to project the potential distribution of *I. scapularis.* ArcGIS software (version 10.5, https://www.arcgis.com/, accessed on 23 May 2021) was used to visualize the projection results. R software (version 4.0.0, http://www.r-project.org/, accessed on 31 May 2021) and DIVA-GIS software (version 7.5.0, https://www.diva-gis.org/, accessed on 31 May 2021) were used to adjust model parameters. The world map (scale 1:10,000,000) was downloaded from the Natural Earth (http://www.naturalearthdata.com, accessed on 13 June 2021). The scale of the map in this article represents mid-latitude distances.

### 2.2. Distribution Data Collection and Processing of I. scapularis

**Figure 1:**
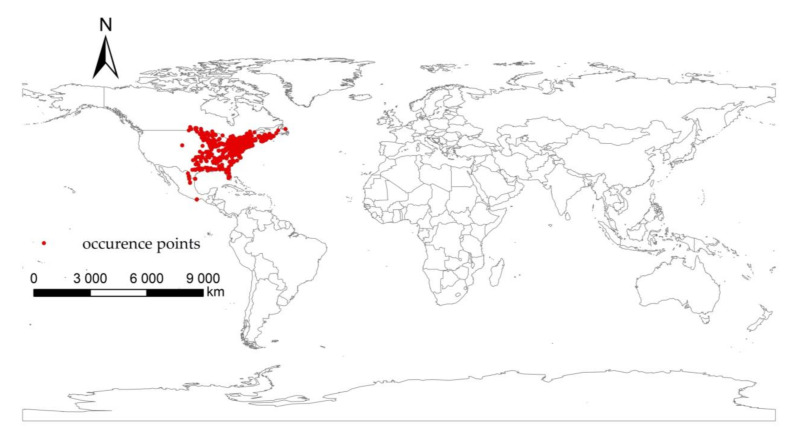
Current occurrence of *Ixodes scapularis*.

The distribution data of *I. scapularis* were obtained from the Global Biodiversity Information Facility (GBIF) (https://www.gbif.org/. accessed on 13 June 2021). Via Google Earth (https://earth.google.com, accessed on 15 June 2021), the accuracy of coordinate points were checked and points with apparent errors in geographical coordinates were removed. To eliminate the possible spatial sampling bias caused by too dense distribution points in a certain region, Proximity in ArcMap was used to create a 2.5 km buffer with points as factors to screen the distribution data. After the above processing, we obtained 1140 points of *I. scapularis*. All sample point data were saved in a CSV file, which contained three columns, i.e., species name, longtitude and latitude coordinate values. It can be seen from the figure that *I. scapularis* is mainly distributed in the eastern part of the United States (Figure 1). 

### 2.3. Environmental Data

The following were downloaded: 19 bioclimatic variables, 36 historical climate data (spatial resolution 2.5 min) of monthly minimum temperature (Tmin1–12), monthly maximum temperature (Tmax1–12), monthly total precipitation (Prec1–12), and elevation from Worldclim v2.1 released in January 2020 (http://worldclim.org/version2, accessed on 16 June 2021) (Table S1). The shared socioeconomic paths (SSPs) of BCC-CSM2-MR, i.e., SSP1-2.6, SSP2-4.5, SSP3-7.0, and SSP5-8.5 (spatial resolution 2.5 min), were simulated as future climate conditions. 

### 2.4. Selection of Model Parameters

Recent studies have shown that the MaxEnt model using default parameters may not be optimal, especially when there are few species distribution points [20]. We need to adjust the parameters to prevent the model from being too complex and/or overfitting [21,22]. Maxent optimizes the model by modifying the two parameters of feature classes (FC) and regularization multiplier (RM). The FC consist of lineal (L), quadratic (Q), product (P), threshold (T), and hinge (H) [23]. The RM parameters are set to eight levels: 0.5, 1, 1.5, 2, 2.5, 3, 3.5, and 4. To avoid overfitting/complexity, we used the R software package “ENMeval” to obtain the best model through Akaike information criterion (AIC). When AICc is the lowest (i.e., AICc = 0), the model is considered to be the most appropriate [24]. The FC and RM of the MaxEnt model in this study were LQHPT and 4.

### 2.5. Project the Potential Suitable Area

The occurrence points and environment variables were imported into the MaxEnt model; 25% distribution points were randomly selected as testing data, and the remaining 75% as training data. The maximum iteration mode was “bootstrap”, and the maximum number of repetitions was 10,000. The number of repeated training data was set to 20 times. The threshold rule was used to select the “10 percentile training presence”. There may be multicollinearity between environmental variables [25]. In order to avoid overfitting the MaxEnt model, we used the Pearson coefficient to test the potential correlation of environmental variables [26]. When Pearson’s |r| > 0.8, it was considered to be highly correlated. According to the contribution of environmental variables, we selected them from high to low, and eliminated the highly related variables. The receiver operating characteristic curve (ROC) was used to test the projection accuracy [18,23]. When the area under curve (AUC) was less than 0.7, the model accuracy was considered to be low; when it was between 0.7 and 0.9, the model accuracy was considered to be medium; and when it was greater than 0.9, the model accuracy is considered to be high [27,28]. The output format was ASCII grid layer, and the range of fitness index was 0–1. The running results of MaxEnt were loaded into ArcMap 10.5 for reclassification and visual expression. The distribution area of *I. scapularis* was divided into unsuitable, high suitable, middle suitable, and low suitable areas by the Jenks natural breaks classification method in ArcGIS. The criteria are: 0–0.07, unsuitable area; 0.08–0.29, low suitable area; 0.30–0.56, middle suitable area; 0.57–1.00, high suitable area.

## 3. Results

### 3.1. The Main Contributing Environmental Variables

**Figure 2:**
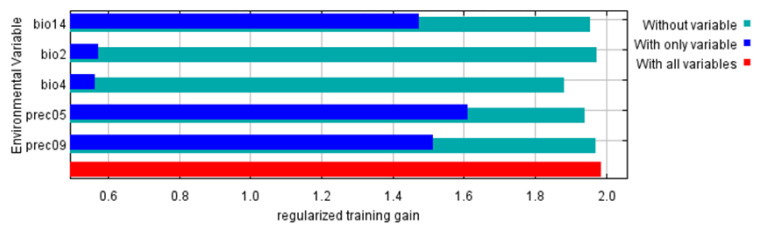
The results of the jackknife test of variable importance.

The geographical distribution of *I. scapularis* was most affected by five climatic factors. They were precipitation in May (Prec05), precipitation in September (Prec09), precipitation of the driest month (Bio14), temperature seasonality (Bio4) and the mean diurnal range (Bio2) (Table 1). Prec05 contains the most useful information, while Bio4 contains the most information not present in the other variables (Figure 2).

### 3.2. The Suitable Areas of I. scapularis under Near Current Climatic Condition

**Figure 3:**
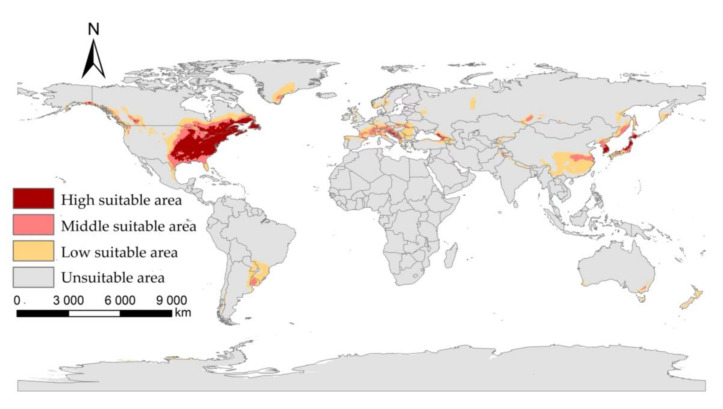
The suitable areas of *I. scapularis* under near current climatic condition.

Under current climatic condition, the suitable areas of the *I. scapularis* in the world are mainly in parts of America, Europe, and Asia. In North America, it is mainly distributed in the eastern United States, the southeast and southwest of Canada, the northeast of Mexico, and the Bahamas. In South America, it is mainly distributed in Uruguay, southern Brazil, northeastern Argentina, and southeastern Paraguay. In Europe, it is mainly distributed in Switzerland, the southern part of France, Italy, Austria, Germany, etc. In Asia, it is mainly distributed in the southeastern part of China, South Korea, and Japan. It is also distributed in New Zealand and southeastern Australia in Oceania (Figure 3). 

### 3.3. The Suitable Areas of I. scapularis under Future Climate Change Scenarios

**Figure 4:**
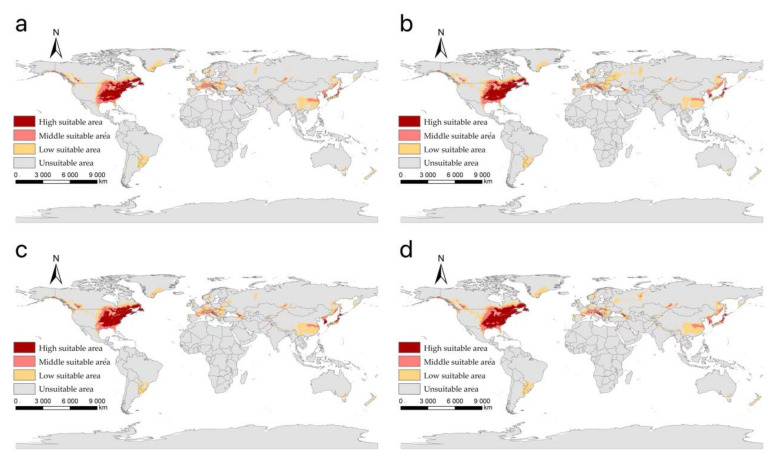
The suitable areas of *I. scapularis* around the world under shared socioeconomic pathway 1–2.6 (sustainability and radiative forcing of 2.6 W/m^2^ to 2100) during different periods of the 21st century: (**a**) 2021–2040; (**b**) 2041–2060; (**c**) 2061–2080; (**d**) 2081–2100.

**Figure 5:**
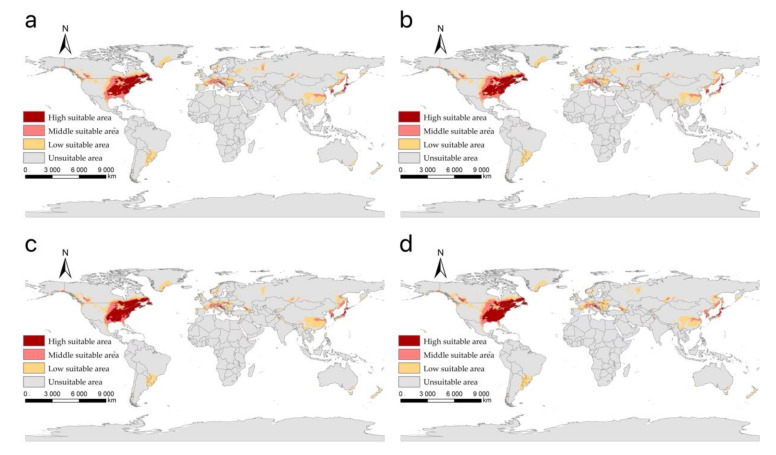
The suitable areas of *I. scapularis* around the world under shared socioeconomic pathway 2–4.5 (middle of the road and forcing of 4.5 W/m^2^ to 2100) during different periods of the 21st century: (**a**) 2021–2040; (**b**) 2041–2060; (**c**) 2061–2080; (**d**) 2081–2100.

**Figure 6:**
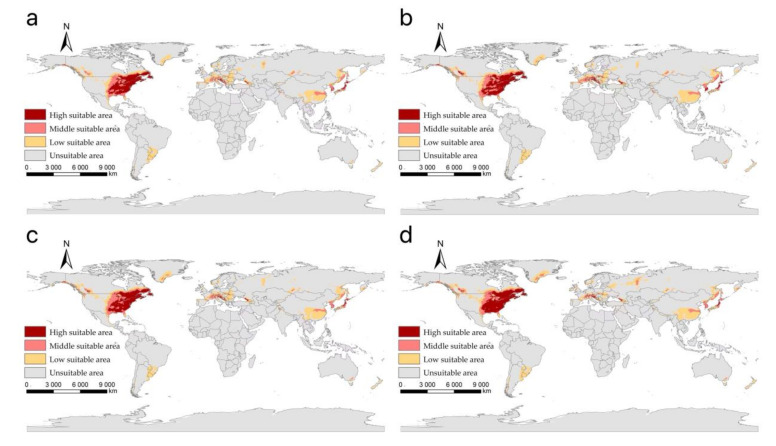
The suitable areas of *I. scapularis* around the world under shared socioeconomic pathway 3–7.0 (regional rivalry and forcing of 7.0 W/m^2^ to 2100) during different periods of the 21st century: (**a**) 2021–2040; (**b**) 2041–2060; (**c**) 2061–2080; (**d**) 2081–2100.

**Figure 7:**
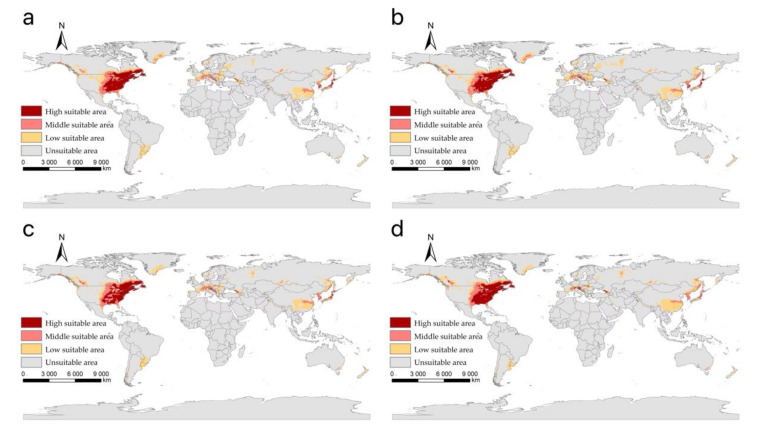
The suitable areas of *I. scapularis* around the world under shared socioeconomic pathway 5–8.5 (fossil-fuel development and forcing of 8.5 W/m^2^ to 2100) during different periods of the 21st century: (**a**) 2021–2040; (**b**) 2041–2060; (**c**) 2061–2080; (**d**) 2081–2100.

**Figure 8:**
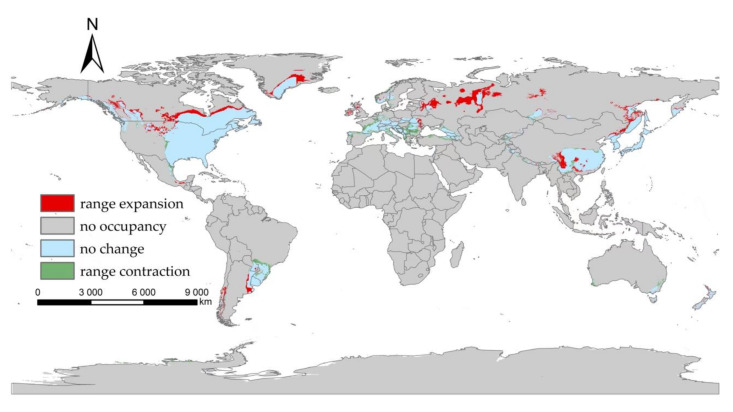
Comparison of the current suitable area with the period 2081–2100 under SSP3-7.0.

**Figure 9:**
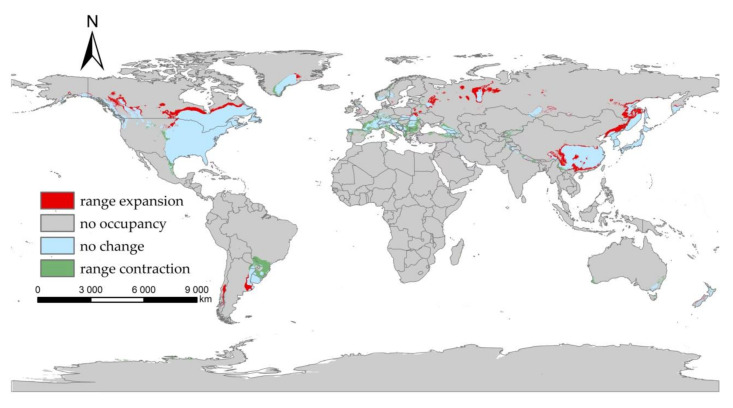
Comparison of the current suitable area with the period 2081–2100 under SSP5-8.5.

Our projected results show a dynamic change in the suitable area of *I. scapularis* around the world from 2021 to 2100 under four future climate change scenarios (Figure 4, Figure 5, Figure 6 and Figure 7). Compared with the current suitable area, the potential suitable area will increase under the scenarios of SSP1-2.6, SSP2-4.5, and SSP3-7.0 (1.17–10.41%), while under SSP5-8.5, the potential suitable areas in the periods 2021–2040 and 2041–2060 will increase (4.92% and 5.66%), but in the periods 2061–2080 and 2081–2100 will decrease (−1.79% and −3.38%) (Table 2). The potential suitable area of *I. scapularis* will be the largest in the period 2081–2100 SSP3-7.0. Compared with the current suitable area, it will expand northward in Canada and Greenland. In addition, western Russia, southwest and northeast China, northeast Argentina, and southeast Chile whill also have significant expansion (Figure 8). In the period 2081–2100 SSP5-8.5, the area of the potential suitable area will be the smallest. Compared with the current suitable area, southern Europe, South America, Brazil, Uruguay, and other regions, as well as central Asia, will have a significant contraction (Figure 9). 

**Figure 10:**
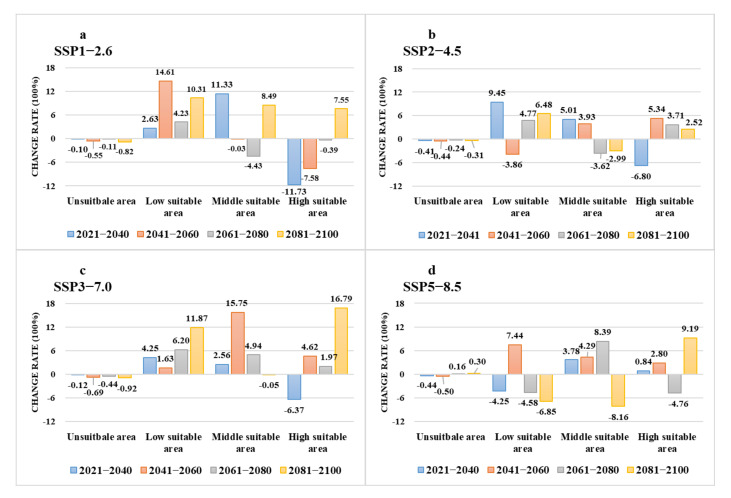
The change rate of future suitable areas as compared with current suitable areas and the four shared socio-economic pathways: (**a**) SSP1-2.6; (**b**) SSP2-4.5; (**c**) SSP3-7.0; (**d**) SSP5-8.5.

Under various climate change scenarios, the minimum high suitable area was projected in the period 2021–2040 SSP1-2.6, reducing to an area of 2.50 × 10^6^ km^2^. Compared with the current high suitable area, it decreased by 11.73% (Figure 10a). Under the SSP3-7.0, the projected high suitable area is the largest from 2081 to 2100, reaching 3.31 × 10^6^ km^2^, an increase of 16.79% over the current climate conditions (Figure 10c). Middle suitable areas show a downward trend under SSP2-4.5 (Figure 10b). Under the SSP5-8.5 scenario, there will be a significant contraction of the low suitability area (Figure 10d).

### 3.4. The Accuracy of the MaxEnt Model

**Figure 11:**
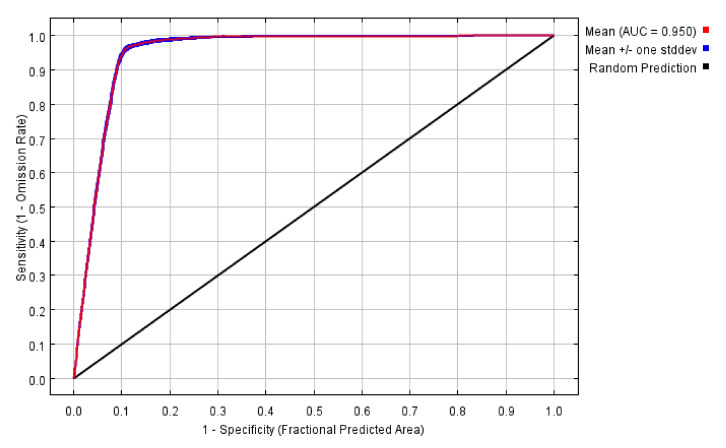
The AUC projected by the MaxEnt model of *I. scapularis*.

The ROC was used to test the projection accuracy. The AUC of the MaxEnt model was 0.950 (Figure 11), and the standard deviation was 0.001, which showed that the accuracy of the model was high.

## 4. Discussion

In the two-year life cycle of *I. scapulalis*, more than 95% of the time is spent outside the host, either looking for the host or diapause [29]. Because of their high surface area to volume ratio, *I. scapulalis* is susceptible to desiccation when questing for hosts [30]. Therefore, *I. scapulalis* needs a suitable habitat to survive and reproduce. We found that the most important environmental variables affecting the potential distribution of *I. scapulalis* were precipitation in May (Prec05), precipitation in September (Prec09), precipitation in the driest month (Bio14), temperature seasonality (Bio4), and the mean diurnal range (Bio2). Micah et al. [31] found that the main environmental variables affecting the distribution of *I. scapulalis* in the USA were the max temperature of the warmest month (Bio5) and precipitation of the warmest quarter (Bio18). *I. scapularis* expand its range into northwestern Minnesota, as well as central and northern Michigan in the USA. Johnson et al. [32] predicted the habitat suitability of *I. scapularis* in Minnesota, in which land cover, temperature annual range (Bio7), and precipitation of the wettest quarter (Bio16) contributed the most. Their model predicted that the habitat of *I. scapularis* extended up into the Minnesota River Valley. Although the main environmental variables derived from this study are slightly different from previous studies, the projected distribution of suitable areas in the Americas is consistent. One possible reason is that the study only included bioclimatic variables, not variables such as monthly total precipitation. The second reason may be that it refers to WorldClim1.4, which is the newer version 2.1 used in this study. Micah et al. [31] and Johnson et al. [32] mainly focused on the Americas, while this study projected the distribution of the global suitable areas of *I. scapularis.* Other studies have shown that Ontario, Canada, will become a high-risk area for *I. scapulalis* in the 2050s [4,33]. This is consistent with our projections.

Dry years may result in reduced nymphal activity and mortality as a result of desiccation [30]. In hot summer, enough precipitation is needed to offset the death of *I. scapulalis* from heat [32]. However, heavy precipitation associated with tropical cyclones is projected to be higher at 2 °C as compared with 1.5 °C of global warming [34], that is, global warming will bring more extreme weather such as droughts and floods [35]. If precipitation and temperature exceed the tolerance of *I. scapulalis*, then, these may be the reasons for the projected potential contraction of distribution areas under the SSP5-8.5 scenario from 2061 to 2100. When studying the ecological factors related to *I. scapulalis*, it is critical to study its microhabitat characteristics [36]. Some data show that soil properties, such as litter depth, can affect the survival and density of *I. scapulalis*. Proper litter and canopy cover are helpful to stabilize the microclimate of ticks [37,38]. 

The distribution of *I. scapularis* is indeed affected by many factors, including vegetation and host. Such variables including soil properties, vegetation types, and the main host distribution are not available in this study, which is a limitation.

In this study, the MaxEnt was used to project the potential distribution of *I. scapulalis*. The accuracy of the model was greater than 0.9 and the fitting was good. The MaxEnt model is popular and has been used by many researchers to predict the distribution of species. In the future, we need more data for simulation scenario analysis to make the MaxEnt model more accurate and reliable. In addition, MaxEnt needs accurate longitude and latitude information, but these can hardly be obtained from the previous data or literature. Subsequent research should consider taking the region as the research object [39] and using the enhanced regression tree model for projection.

With global climate change, the potential distribution of *I.scapulalis* is also changing. The projection results show that some areas such as Greenland and southern Chile have suitable areas, that is, the potential suitable areas of *I.scapulalis* is expanding to higher latitudes. Climate change is indeed affecting the spread of vector-borne infectious diseases, so we should actively respond to climate change and reduce the harm brought by climate change to human beings. 

## 5. Conclusions

We used climate conditions and occurrence points to project the potential distribution of *I.scapulalis* under MaxEnt. It is found that the range of suitable areas generally showed an upward trend under the future climate change scenarios, and the largest area is in the periods 2081–2100 SSP3-7.0, but the suitable area of SSP5-8.5 shows a downward trend in the periods 2061–2080 and 2081–2100. Precipitation and temperature are the main factors affecting the distribution of suitable areas. With global climate change, the potential suitable areas of *I.scapulalis* is likely expanding to higher latitudes. Climate change is indeed affecting the distribution of ticks’ suitable areas.. It is critical to actively respond to climate change.

## Figures and Tables

**Table 1:** The high contributing environmental variables of the potential suitable area of *Ixodes scapularis*.

Variables	Description	Unit	Contribution (%)
Prec05	Precipitation in May	mm	39.6
Prec09	Precipitation in September	mm	22.0
Bio14	Precipitation of the driest month	mm	19.6
Bio4	Temperature seasonality (standard deviation × 100)	\	17.0
Bio2	Mean diurnal range (mean of monthly (max temp—min temp))	°C	1.80

**Table 2:** Current and future suitable areas of *I. scapularis* under different climatic conditions (×10^6^ km^2^).

Climate Scenario	Period	Low Suitable Area	Middle Suitable Area	High Suitable Area	Total Area	Area Change	Area Change Ratio (%)
current	1970–2000	6.63	2.64	2.83	12.10	0.00	
SSP1-2.6	2021–2040	6.80	2.94	2.50	12.24	0.14	1.17
	2041–2060	7.59	2.64	2.62	12.86	0.75	6.21
	2061–2080	6.91	2.53	2.82	12.26	0.15	1.25
	2081–2100	7.31	2.87	3.05	13.22	1.12	9.26
SSP2-4.5	2021–2040	7.25	2.78	2.64	12.67	0.57	4.67
	2041–2060	6.97	2.75	2.98	12.70	0.60	4.97
	2061–2080	6.94	2.55	2.94	12.43	0.33	2.69
	2081–2100	7.06	2.56	2.90	12.53	0.42	3.48
SSP3-7.0	2021–2040	6.91	2.71	2.65	12.27	0.17	1.39
	2041–2060	7.02	3.06	2.96	13.04	0.94	7.77
	2061–2080	7.04	2.77	2.89	12.70	0.60	4.93
	2081–2100	7.41	2.64	3.31	13.36	1.26	10.41
SSP5-8.5	2021–2040	7.10	2.74	2.86	12.70	0.60	4.92
	2041–2060	7.12	2.76	2.91	12.79	0.69	5.66
	2061–2080	6.32	2.87	2.70	11.89	−0.22	−1.79
	2081–2100	6.17	2.43	3.09	11.69	−0.41	−3.38

## Data Availability

Environmental variables can be downloaded from Worldclim v2.1 released in January 2020 (http://worldclim.org/version2, accessed on 16 June 2021). Occurrence records can be downloaded from GBIF (https://www.gbif.org, accessed on 13 June 2021).

## Supplementary Materials

The following supporting information can be downloaded at: https://www.mdpi.com/article/10.3390/biology11010107/s1, Table S1: Climate variables used in projecting the potential geographic distribution of *I. scapularis*, Table S2: The distribution points of *I. scapulalis* for model operation.
