# Supplementary material for: Projecting the Potential Distribution Areas of Ixodes scapularis (Acari: Ixodidae) Driven by Climate Change
Source: Biology (Basel). 2022 Jan 10;11(1):107. doi: 10.3390/biology11010107 (PMC8773098; doi:10.3390/biology11010107)
Supplement: Supplementary file 1 [file biology-11-00107-s001.zip › Table S1 Climate variables used in projecting the potential geographic distribution of I. scapularis..pdf]

Table S1. Climate variables used in projecting the potential geographic distribution of *I. scapularis.*

Code	Climate Variables
Bio1	Annual Mean Temperature
Bio2	Mean Diurnal Range (Mean of monthly (max temp – min temp))
Bio3	Isothermality (Bio2/Bio7) (*100)
Bio4	Temperature Seasonality (standard deviation*100)
Bio5	Max Temperature of Warmest Month
Bio6	Min Temperature of Coldest Month
Bio7	Temperature Annual Range (Bio5 – Bio6)
Bio8	Mean Temperature of Wettest Quarter
Bio9	Mean Temperature of Driest Quarter
Bio10	Mean Temperature of Warmest Quarter
Bio11	Mean Temperature of Coldest Quarter
Bio12	Annual Precipitation
Bio13	Precipitation of Wettest Month
Bio14	Precipitation of Driest Month
Bio15	Precipitation Seasonality (Coefficient of Variation)
Bio16	Precipitation of Wettest Quarter
Bio17	Precipitation of Driest Quarter
Bio18	Precipitation of Warmest Quarter
Bio19	Precipitation of Coldest Quarter
Tmin1-12	Monthly Average Minimum Temperature
Tmax1-12	Monthly Average Maximum Temperature
Prec1-12	Monthly Total Precipitation
Elevation	Elevation data
